# GC-MS analysis and screening of antidiabetic, antioxidant and hypolipidemic potential of *Cinnamomum tamala* oil in streptozotocin induced diabetes mellitus in rats

**DOI:** 10.1186/1475-2840-11-95

**Published:** 2012-08-10

**Authors:** Suresh Kumar, Neeru Vasudeva, Sunil Sharma

**Affiliations:** 1 Pharmacology Division, Department of Pharmaceutical Sciences, Guru Jambheshwar University of Science and Technology, Post Box: 38, Hisar, 125001, India; 2 Pharmacognosy Division, Department of Pharmaceutical Sciences, Guru Jambheshwar University of Science and Technology, Post Box: 38, Hisar, 125001, India

**Keywords:** Cinnamomum tamala, Cinnamaldehyde, Glibenclamide, Hyperglycaemia, Streptozotocin

## Abstract

**Aim of the study:**

This study was made to investigate the antidiabetic, antioxidant and hypolipidemic potential of *Cinnamomum tamala*, (Buch.-Ham.) Nees & Eberm (Tejpat) oil (CTO) in streptozotocin (STZ) induced diabetes in rats along with evaluation of chemical constituents.

**Materials and methods:**

The GC-MS (Gas chromatography–mass spectrometry) analysis of the oil showed 31 constituents of which cinnamaldehyde was found the major component (44.898%). CTO and cinnamaldehyde was orally administered to diabetic rats to study its effect in both acute and chronic antihyperglycemic models. The body weight, oral glucose tolerance test and biochemical parameters viz. glucose level, insulin level, liver glycogen content, glycosylated hemoglobin, total plasma cholesterol, triglyceride and antioxidant parameters were estimated for all treated groups and compared against diabetic control group.

**Results:**

CTO (100 mg/kg and 200 mg/kg), cinnamaldehyde (20 mg/kg) and glibenclamide (0.6 mg/kg) in respective groups of diabetic animals administered for 28 days reduced the blood glucose level in streptozotocin induced diabetic rats. There was significant increase in body weight, liver glycogen content, plasma insulin level and decrease in the blood glucose, glycosylated hemoglobin and total plasma cholesterol in test groups as compared to control group. The results of CTO and cinnamaldehyde were found comparable with standard drug glibenclamide. *In vitro* antioxidant studies on CTO using various models showed significant antioxidant activity. *In vivo* antioxidant studies on STZ induced diabetic rats revealed decreased malondialdehyde (MDA) and increased reduced glutathione (GSH).

**Conclusion:**

Thus the investigation results that CTO has significant antidiabetic, antioxidant and hypolipidemic activity.

## Introduction

Diabetes mellitus is one of the major global health and economic problem, characterized by high levels of blood glucose resulting from defects in insulin production, insulin action, or both. Diabetes has affected 6% of the world’s population [1,2]. Type II diabetes accounts for 90–95% of all diabetic cases [2]. Long-term complications viz; cardiomyopathy, angiopathy, nephropathy etc. are a major cause of morbidity in patients with diabetes mellitus. Hyperlipidemia and oxidative stress frequently co-exist with diabetes mellitus [3]. The increased blood glucose levels in diabetes produce superoxide anions, which generate hydroxyl radicals via Haber Weiss reaction, resulting in peroxidation of membrane lipids and protein glycation causing oxidative damage of cell membranes. These radicals further damage other important biomolecules including carbohydrates, proteins and deoxyribonucleic acid (DNA) [4]. Antioxidants play an important role to protect the human body against damage caused by reactive oxygen species. Hence compounds with both hypoglycemic and antioxidant properties would be useful antidiabetic agent.

Some studies have suggested that essential oils may be useful in the treatment of insulin resistance and type II diabetes mellitus, and various oils have been used as therapeutic agents for years without any significant adverse health effects. *Cinnamomum tamala* (Buch.-Ham.) Nees & Eberm (Tejpat) (Lauraceae), a volatile oil containing tree is commercially known as Indian cassia. It is used in traditional medicines as an astringent, stimulant, diuretic, carminative and in cardiac disorders [5]. The leaves of *Cinnamomum tamala* have been reported to possess antidiabetic, antioxidant [6], antidiarrhoeal [7], antihyperlipidemic [8], antioxygenic [9], anti-inflammatory [10], acaricidal [11], hepatoprotective [12], gastroprotective [13], antibacterial and immunomodulatory activities [14]. The essential oil from *Cinnamomum* species can be extracted easily by hydro distillation [15]. The oil has been widely used as a flavoring agent and additives for centuries in the food industries. As far as we know, the effect of oil on the blood profiles in diabetic models has not been studied. In light of these findings, we carried out this study for the evaluation of antidiabetic, hypolipidemic and antioxidant potential of the CTO.

## Materials and methods

### Drugs and chemicals

The drugs and chemicals used in the study were glibenclamide (Torrent Pharmaceutical, Ahmadabad), streptozotocin, heparin (SRL, India), EDTA (Hi-media Lab. Pvt Ltd., Mumbai, India), Ellman’s reagent (5,5’-dithiobis-(2-nitro-benzoic acid); DTNB), sodium sulphate, methanol, pyridine, anthrone, thiourea, benzoic acid, sodium chloride (SD Fine Chem Ltd., Mumbai, India). All the chemicals used in the study were of analytical grade.

### Preparation of oil

The dried leaves *of Cinnamomum tamala* procured from local market of Hisar which were identified and authenticated by Dr. H. B. Singh, Head, Raw Materials Herbarium and Museum, National Institute of Science Communication and Information Resources (Ref. NISCAIR/RHMD/Consult/-2011-12/1858/158), Delhi (India). The leaves were cut in to small pieces and oil was extracted with the help of Clevenger apparatus. The percentage yield of the oil was found to be 0.45%.

### Gas chromatography–mass spectrometry (GC-MS) analysis

The GC-MS analysis of the essential oil was performed using Agilent 7890A GC system equipped with MS detector 5975C inert XL EI/CI MSD having automatic sampler CTC analysis CombiPAL robotic arm. For GC/MS detection, an electron ionization system with ionization energy of 70 eV was used. Helium gas was used as the carrier gas at a constant flow rate of 1 ml/min. The inlet temperature was set at 270°C. The specification of the capillary column used was Agilent 19091S-433: 1548, 52849 HP-5MS 5% Phenyl Methyl Silox 30 m × 250 μm x 0.25 μm HP-5MS. The oven temperature was programmed from 80°C to 300°C. The diluted samples (1/100, v/v, in Hexane) of 2 μL were injected.

### Identification of constituents

The relative percentage amount of each component was calculated by comparing its average peak area to the total areas. The oils components were identified by matching their recorded mass spectra with the data bank mass spectra (Search library Database/W9N08.L) and by comparing their retention indices relative to a series of n-hydrocarbons (C7–C23) with literature values [16].

### Experimental animals

Healthy male albino wistar rats (150–250 g, 60–90 days old) were procured from Disease Free Small Animal House, Chaudhary Charan Singh Haryana Agriculture University, Hisar (Haryana). The rats were housed in (Polycarbonate cage size: 29 × 22 × 14 cm) under laboratory standard conditions (25 ± 3°C:35–60% humidity) with alternating light and dark cycle of 12 h each and were feed fed with a standard rat pellet diet (Hindustan Lever Ltd, Mumbai, India) and water *ad libitum*. The experimental protocol was approved by Institutional Animals Ethics Committee (IAEC) and animal care was taken as per the guidelines of Committee for the Purpose of Control and Supervision of Experiments on Animals (CPCSEA), Govt. of India (Registration No. 0436).

### Acute toxicity studies

Healthy adult albino wistar rats of both sex, starved overnight were divided in to eight groups (n = 6) and were orally fed with the oil of *Cinnamomum tamala* in the increasing dose of 10, 50, 100, 200, 500, 1000, 1500 and 2000 mg/kg body weight. The rats were observed continuously for 2 h for behavioral changes and after 24 and 72 h for any lethality [17].

### Induction of diabetes

Type II diabetes mellitus (NIDDM) was induced in overnight fasted animals by a single intraperitoneal injection of 50 mg/kg STZ in 0.1 M citrate buffer (pH-4.5) in a volume of 1 ml/kg body weight. Diabetes was developed and stabilized over a period of 7 days. Diabetes was confirmed by the elevated blood glucose levels determined at 72 h and on 7th day after injection. Only rats confirmed with permanent NIDDM were used in the antidiabetic study. Blood was collected by intraocular route [18].

### Experimental design

After the induction and confirmation of diabetes, Rats were divided into the following groups comprising six rats in each group.

### For acute antihyperglycemic model

In the acute antihyperglycemic models the study was carried out for 4 hours to check whether the plant have some effect or not.

Group 1 Normal rats were administered 2% Dimethyl sulfoxide (DMSO).

Group 2 Diabetic control rats were administered 2% Dimethyl sulfoxide (DMSO).

Group 3 Diabetic animals were administered glibenclamide (0.6 mg/kg p.o).

Group 4 Diabetic animal were administered orally 100 mg/kg of CTO.

Group 5 Diabetic animal were administered orally 200 mg/kg of CTO.

Group 6 Diabetic animal were administered orally 20 mg/kg of Cinnamaldehyde.

### For chronic antihyperglycemic model

In the chronic antihyperglycemic models the study was carried out for 28 days to study the various parameters of the diabetes and hyperlipidemia to confirm the antidiabetic, antioxidant and hypolipidemic activity of *Cinnamomum tamala* oil and its main constituent cinnamaldehyde in streptozotocin induced diabetes in rats.

Group 7 Normal rats were administered 2% Dimethyl sulfoxide (DMSO).

Group 8 Diabetic control rats were administered 2% Dimethyl sulfoxide (DMSO).

Group 9 Diabetic animals were administered glibenclamide (0.6 mg/kg p.o).

Group 10 Diabetic animal were administered orally 100 mg/kg of CTO.

Group 11 Diabetic animal were administered orally 200 mg/kg of CTO.

Group 12 Diabetic animal were administered orally 20 mg/kg of Cinnamaldehyde.

### Sample collection

#### Blood sample

The 24 h fasted animals were sacrificed by cervical decapitation on 29th day of treatment. Trunk blood was collected in heparinized tubes and the plasma was obtained by centrifugation at 5000 rpm for 5 min. for the determination of biochemical parameters; glucose, insulin, cholesterol, glycosylated hemoglobin, malondialdehyde (MDA), reduced glutathione (GSH) etc.

#### Collection of organs

The rats were anaesthetized by using the overdose of anesthesia, and tissue sample were taken for assessment of biochemical parameters.

### Estimation of plasma glucose and cholesterol

Plasma cholesterol and glucose level were measured by commercial supplied biological kit Erba Glucose Kit (GOD-POD Method) and Erba Cholesterol Kit (CHOD-PAP Method) respectively using Chem 5 Plus-V_2_ Auto-analyser (Erba Mannhein Germany) in plasma sample prepared as above. Glucose and cholesterol values were calculated as mg/dl blood sample.

### Estimation of glycosylated hemoglobin (Hb1Ac)

Glycosylated hemoglobin was measured using commercial supplied biological kit (Erba Diagnostic) in plasma sample prepared as above using Chem 5 Plus-V_2_ Auto-analyser (Erba Mannhein Germany). Values are expressed as the percent of total hemoglobin.

### Estimation of liver glycogen content

Liver glycogen estimation was done by the method as described by Seifter *et al.* (1950) [19]. Immediately after excision from the animal, 1 g of the liver was dropped into a previously weighed test tube containing 3 ml of 30% potassium hydroxide solution. The weight of the liver sample was determined. The tissue was then digested by heating the tube for 20 min in boiling water bath, and following this the digest was cooled, transferred quantitatively to a 50 ml volumetric flask, and diluted to the mark with water. The contents of the flask were then thoroughly mixed and a measured portion was then further diluted with water in a second volumetric flask so as to yield a solution of glycogen of 3–30 μg/ml. Five ml aliquots of the final dilution were then pipette into Evelyn tube and the determination with anthrone was carried out. The amount of glycogen in the aliquot used was then calculated using the following equation:

(1) μgofglycogeninaliquot=100U/1.11S

U is the optical density of unknown solution. S is the optical density of the 100 μg glucose and 1.11 is the factor determined by Morris in 1948 for the conversion of the glucose to the glycogen.

### Serum insulin assay by ELISA kit

Serum insulin level was measured by an enzyme-linked immunosorbent assay (ELISA) procedure using Mercodia rat insulin ELISA kit. Briefly, the solid phase two-site enzyme immunoassay is based on the direct sandwich technique in which two monoclonal antibodies are directed against separate antigenic determinants 35 (epitopes) on the insulin molecule. During incubation, insulin in the sample reacts with peroxidase-conjugated anti-insulin antibodies and anti-insulin antibodies bound to the micro titration well. After washing three times, unbound enzyme labeled antibody was removed. The bound conjugated insulin was detected by reacting with 3, 3’, 5, 5’-tetramethylbenzidine. The reaction was stopped by adding acid to give a colorimetric end-point and optical density was measured with a micro plate auto reader (Bio-tek Instrument Inc., USA) at a wavelength of 450 nm. The serum insulin is expressed as μg/l.

## *In vitro* antioxidant activity

### Diphenyl-picryl-hydrazyl radical scavenging (DPPH) Assay

The antioxidant activity of the oil was measured in terms of hydrogen donating or radical scavenging ability, using the stable radical, DPPH [20]. A methanolic stock solution (50 ml) of the antioxidant (concentrations of stock solutions were 1.0, 2.0, 4.0, 6.0, 8.0, 10, 12, 16, 20.0, 25, 30, 40.0, 45.0 and 50.0 g/l) was placed in a cuvette, and 2 ml of 6 × 10^-5^ M methanolic solution of DPPH was added. Absorbance measurements commenced immediately. The decrease in absorbance at 517 nm was determined by Perkin-Elmer spectrophotometer after 1 h for all samples. Methanol was used to zero the spectrophotometer. The absorbance of the DPPH radical without antioxidant, i.e. the control, was measured daily. Special care was taken to minimize the loss of free radical activity of the DPPH radical stock solution [21]. All determinations were performed in triplicate. The percentage inhibition of the DPPH radical by the samples was calculated according to the formula of Yen and Duh (1994) [22]:

The percentage of inhibition was calculated using the formula,

(2) $$Inhibition\;\left(\%\right)=\left(absorbance\;of\;control–absorbance\;of\;test/absorbance\;of\;control\right)\times 100$$

### Hydrogen peroxide radical scavenging (H2O2) assay

The ability of plant extracts to scavenge hydrogen peroxide is determined according to the method of Ruch et al. (1989) [23]. A solution of hydrogen peroxide (40 mM) was prepared in phosphate buffer (50 mM, pH 7.4). The concentration of hydrogen peroxide was determined by absorption at 230 nm using a spectrophotometer. The oil (20–60 μg/ml) in distilled water was added to hydrogen peroxide and absorbance at 230 nm was determined after 10 min against a blank solution containing phosphate buffer without hydrogen peroxide. The percentage of hydrogen peroxide scavenging was calculated as follows:

(3) $$\%\;Scavenged\;\left(H_{2}O_{2}\right)=\left(A_{0}–A_{1}/A_{0}\right)\times 100$$

Where; A_0_ is the absorbance of control and A_1_ is the absorbance of test. Butylated hydroxy toluene (BHT) was used as a positive control.

### Metal chelating activity

Ferrozine can quantitatively chelate with Fe^2+^ and form a complex with a red color. This reaction is limited in the presence of other chelating agents and results in a decrease of the red color of the ferrozine-Fe^2+^ complexes. Measurement of the color reduction estimates the chelating activity to compete with ferrozine for the ferrous ions [24]. The chelation of ferrous ions is estimated using the method of Dinis et al. (1994) [25]. The oil (0.1 ml) was added to a solution of 0.5 ml ferrous chloride (0.2 mM). The reaction was initiated by the addition of 0.2 ml of ferrozine (5 mM) and incubated at room temperature for 10 min and then the absorbance measured at 562 nm. Ascorbic acid was used as a positive control.

## *In vivo* antioxidant activity

### Estimation of MDA level

Malondialdehyde (MDA), an index of free radical generation/lipid peroxidation, was determined as described by Okhawa *et al.* 1979 [26]. Briefly, the reaction mixture consisted of 0.2 ml of 8.1% sodium lauryl sulphate, 1.5 ml of 20% acetic acid (pH 3.5) and 1.5 ml of 0.8% aqueous solution of thiobarbituric acid added to 0.2 ml of blood plasma. The mixture was made up to 4.0 ml with distilled water and heated at 95°C for 60 min. After cooling the contents under running tap water, 5.0 ml of n-butanol and pyridine (15:1 v/v) and 1.0 ml of distilled water was added. The contents were centrifuged at about 3000 rpm for 10 min. The organic layer was separated out and its absorbance was measured at 532 nm using double beam UV-Visible spectrophotometer (Systronics 2203, Bangalore, India) against a blank. MDA values were calculated using the extinction coefficient of MDA-thiobarbituric acid complex 1.56 × 10^5^ l/mol × cm and expressed as nmol/ml.

### Estimation of plasma reduced glutathione level

The tissue sample (liver 200 mg) was homogenized in 8.0 mL of 0.02 M EDTA in an ice bath. The homogenates were kept in the ice bath until used. Aliquots of 5.0 mL of the homogenates were mixed in 15.0 mL test tubes with 4.0 mL distilled water and 1.0 mL of 50% trichloroacetic acid (TCA). The tubes were centrifuged for 15 min at approximately 3000 rpm, 2.0 mL of supernatant was mixed with 4.0 ml of 0.4 M Tris buffer pH 8.9, 0.1 mL Ellman’s reagent [5,5-dithiobis-(2-nitro-benzoic acid)] (DTNB) added and the sample shaken. The absorbance was read within 5 min of the addition of DTNB at 412 nm against a reagent blank with no homogenate. Results are expressed as μmol GSH/g tissue [27].

### Statistical analysis

The data for various biochemical parameters were evaluated by use of one-way ANOVA, followed by Dunnett’s *t*-test using the software Sigma-Stat 3. In all the tests, the criterion for statistical significance was p <0.05.

## Results

### Chemical composition of essential oil

**Figure 1:**
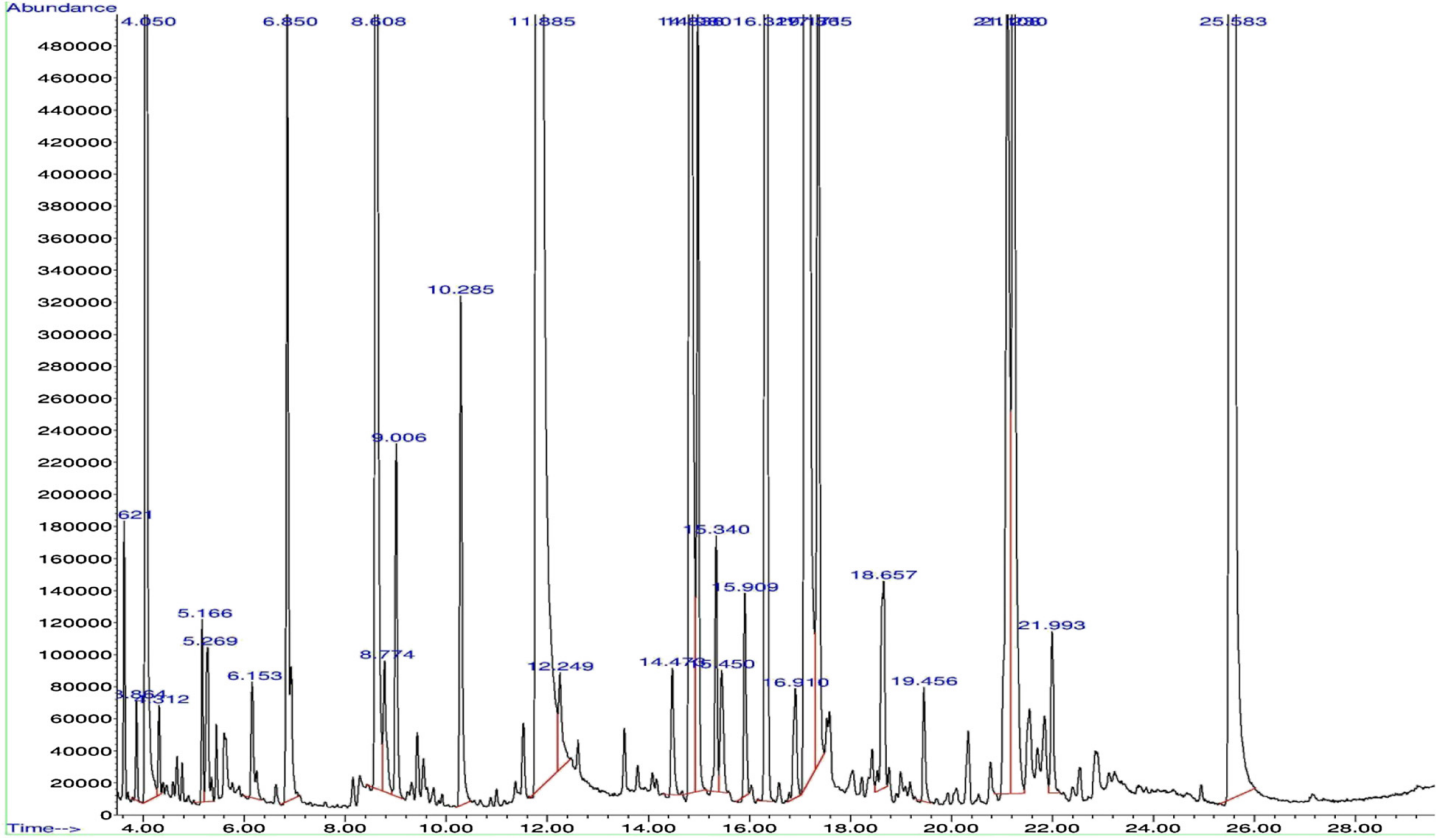
**GC-MS chromatogram of** *** Cinnamomum tamala *** **oil shows 31 peaks which indicates that 31 compounds are present in the plant oil.**

The chromatogram of CTO by GC-MS is shown in Figure 1. The GC-MS analysis of CTO led to the identification and quantification of 31 components (Table 1) which accounted for 99.99% of the total oil. The main volatile components of CTO were found as cinnamaldehyde (44.898%), *Trans* cinnamyl acetate (25.327%), Ascabin (15.249%), Hydro cinnamyl acetate (3.384%), Beta-caryophyllene (2.669%) which comprised of 91.527% of the oil.

**Table 1:** **Chemical composition of** *** Cinnamomum tamala *** **essential oil Total components of the oil 99.99%**

**Sr. No.**	**Compound**	**Retention time**	**% of Total**
1	Alpha-pinene	3.621	0.095%
2	Camphene	3.864	0.037%
3	Benzaldehyde	4.050	1.222%
4	Beta-pinene	4.312	0.034%
5	p-cymene	5.166	0.065%
6	Beta-Phellandrene	5.270	0.106%
7	Acetophenone	6.153	0.044%
8	Linalool	6.850	0.442%
9	Beta-phenylpropionaldehyde	8.608	1.856%
10	Phenetol	8.775	0.109%
11	Benzofuran	9.006	0.185%
12	Acrolein	10.285	0.286%
**13**	**Cinnamaldehyde**	**11.886**	**44.898%**
14	2-propenal, 3-phenyl cinnamaldehyde	12.249	0.087%
15	Eugenol	14.473	0.078%
16	Hydro cinnamyl acetate	14.836	3.384%
17	Alpha-copaene	14.980	0.414%
18	Pivalic acid	15.340	0.129%
19	Cinnamyl acetate	15.450	0.091%
20	Methyl eugenol	15.909	0.107%
21	Beta-caryophyllene	16.329	2.669%
22	Valecene	16.910	0.089%
**23**	***Tans*-cinnamyl acetate**	**17.172**	**25.327%**
24	Alpha-humulene	17.365	0.636%
25	Bicyclogermacrene-lepdozene	18.658	0.191%
26	Naphthalene	19.455	0.067%
27	Spathulenol	21.108	0.780%
28	Caryophyllene oxide	21.230	1.135%
29	Alpha-patchoulene	21.547	0.090%
30	Humulene oxide	21.993	0.097%
**31**	**Ascabin**	**25.584**	**15.249%**

### Acute toxicity study

The oral administration of graded dose of CTO to the rats in our acute toxicity study was found to be non lethal up to the dose of 1000 mg/kg body weight. However 2000 mg/kg body weight of CTO caused 50% motility in the animals.

### Oral glucose tolerance test

The effect of CTO on plasma glucose level after glucose loading of 2 g/kg body weight orally to the diabetic rats is expressed in the Table 2. The blood glucose level rises to a maximum in 60 min after glucose loading. The oil (100 mg/kg and 200 mg/kg body weight) and cinnamaldehyde (20 mg/kg body weight) treated groups showed a significant decrease in level of glucose as compared to control group. The oil treated group showed a marked fall in glucose level in 90 min to 120 min interval.

**Table 2:** **Effect of** *** Cinnamomum tamala *** **oil in Oral glucose tolerance test (OGTT)**

**Treatment**	**Dose**	**Mean blood glucose concentration (mg/dl) ± S.E.M**				
		**0 min.**	**30 min.**	**60 min.**	**90 min.**	**120 min.**
Normal	----	85±4.6	147 ±2.3	110 ± 2.9	97 ± 3.5	89 ± 3.0
Diabetic control	----	270 ± 8.6	399 ± 6.3	419.2 ± 3.2	369 ± 3.6	341 ± 4.9
CTO	100 mg/kg	255 ± 4.8	279 ± 3.9	318 ± 4.0	299 ± 5.6**	280 ± 3.2**
CTO	200 mg/kg	257 ± 4.9	282 ± 4.1	343 ± 3.9	286 ± 2.5**	239 ± 3.8**
Cinnamaldehyde	20 mg/kg	252 ± 4.0	287 ± 5.7	356 ± 2.1	275 ± 4.2**	228 ± 2.9**

Values are presented as mean ± S.E.M.; n = 6 in each group. One way ANOVA followed by *Dunnett’s* test **p < 0.01 vs. diabetic control; *CTO*: *Cinnamomum tamala* oil.

### Effect of CTO on diabetic rats in acute study

Administration of CTO at a dose 100 mg/kg body weight p. o. to diabetic rats showed reduction in blood glucose level from 356 mg/dl to 292 mg/dl at 4^th^ h. When the dose was increased as 200 mg/kg then the blood glucose level decreased from 347 mg/dl to 272 mg/dl which was found significant (p < 0.01) when compared with diabetic control. The main constituent cinnamaldehyde also showed the decrease in blood glucose level significantly (p < 0.01) when compared with the diabetic control groups (Table 3).

**Table 3:** **Acute hypoglycaemic effect of** *** Cinnamomum tamala *** **oil on STZ induced diabetic rats**

**Treatment**	**Dose**	**Mean blood glucose concentration (mg/dl) ± S.E.M)**				
		**0 h**	**1/2 h**	**1 h**	**2 h**	**4 h**
Normal	--	76 ± 4.2	80 ± 3.2	77 ± 2.5	82 ± 4.1	79 ± 5.3
Control	--	340.5 ± 10.2	342 ± 11.3	346 ± 7.6	341.0 ± 6.7	332.0 ± 7.2
CTO	100 mg/kg p.o	356 ± 6.7	334.1 ± 6.2	308 ± 3.6**	301 ± 3.0**	292 ± 3.3**
CTO	200 mg/kg p.o	347 ± 1.8	324 ± 0.7*	301 ± 2.3**	295 ± 1.5**	272 ± 6.5**
Cinnamaldehyde	20 mg/kg p.o	329 ± 3.2	302 ± 4.2**	299 ± 3.2**	270 ± 4.6**	262 ± 5.3**
Glibenclamide	0.6 mg/kg p.o	334 ± 3.1	320 ± 2.9**	296 ± 3.9**	279 ± 4.9**	260.0 ± 4.2**

Values are presented as mean ± S.E.M.; n = 6 in each group. One way ANOVA followed by *Dunnett’s* test *p < 0.05; **p < 0.01 vs. diabetic control; *CTO*: *Cinnamomum tamala* oil.

### Effect of CTO on diabetic rats in chronic study

In chronic study administration of CTO at the dose of 100 mg/kg body weight to diabetic rats for 28 days showed a fall in plasma glucose level from 304 mg/dl to 201 mg/dl on 29^th^ day when compared to 0 day value. CTO at the dose of 200 mg/kg body weight showed a significant (p < 0.01) fall in plasma glucose level from 312 mg/dl to 118 mg/dl on 29^th^ day (Table 4).

**Table 4:** **Chronic hypoglycaemic effect of** *** Cinnamomum tamala *** **oil on STZ induced diabetic rats**

**Treatment**	**Dose**	**Mean blood glucose concentration (mg/dl) ± S.E.M**				
		**0**^**th**^**Day**	**7**^**th**^**Day**	**14**^**th**^**Day**	**21**^**st**^**Day**	**28**^**th**^
Normal	--	80 ± 4.2	79 ± 3.2	82 ± 2.5	85.5 ± 4.1	78 ± 2.1
Control	--	380 ± 7.3	379 ± 7.6	384 ± 6.7	416 ± 7.2	410 ± 5.4
CTO	100 mg/kg p.o	304 ± 12.4	290 ± 8.6**	276 ± 7.8**	250 ± 9.9**	201 ± 10.2**
CTO	200 mg/kg p.o	312 ± 5.7	269 ± 6.3**	176 ± 5.5**	146 ± 4.5**	118 ± 5.5**
Cinnamaldehyde	20 mg/kg p.o	334 ± 3.4	287 ± 4.2**	188 ± 3.2**	159 ± 4.6**	124 ± 3.2**
Glibenclamide	0.6 mg/kg p.o	328 ± 3.1	265 ± 2.9**	172 ± 3.9**	142 ± 4.9**	117 ± 2.7**

Values are presented as mean ± S.E.M.; n = 6 in each group. One way ANOVA followed by *Dunnett’s* test **p < 0.01 vs. diabetic control; *CTO*: *Cinnamomum tamala* oil.

### Effect of CTO on body weight

An increase in the body weight of normal rats was observed whereas the weight of diabetic control rats decrease from day 1 to day 29. CTO at the dose of 200 mg/kg body weight when administered to diabetic rats showed a significant (p < 0.01) decrease in body weight as compared to the diabetic control group. Cinnamaldehyde and glibenclamide also showed significant decrease (p < 0.01) in body weight (Table 5).

**Table 5:** **Effect of** *** Cinnamomum tamala *** **oil on body weight**

**Sr. No.**	**Treatment**	**Dose**	**Initial body weight (g)**	**Final body weight (g)**	**Change in weight**
1.	Normal	--	220 ± 1.1	235 ± 1.5	+15
2.	Diabetic Control	--	215 ± 1.8	195 ± 2.0	−20^a^
4.	CTO	100 mg/kg p.o	230 ± 2.2	225 ± 1.0	−5
5.	CTO	200 mg/kg p.o	220 ± 1.3	210 ± 1.2	−10**
6.	Cinnamaldehyde	20 mg/kg p.o	230 ± 2.0	225 ± 1.4	−05**
7.	Glibenclamide	0.6 mg/kg p.o	220 ± 1.8	215 ± 1.1	−05**

Values are presented as mean ± S.E.M.; n = 6 in each group. One way ANOVA followed by *Dunnett’s* test ^a^ p < 0.01 vs. normal; **p < 0.01 vs. diabetic control; *CTO*: *Cinnamomum tamala* oil.

### Effect of CTO on insulin level

Table 6 shows the level of plasma insulin in the control and experimental groups of rats. Diabetic rats showed a significant decrease in plasma insulin compared with normal rats. Following dose of oral administration of CTO, cinnamaldehyde and glibenclamide, plasma insulin levels increased when compared to control rats.

**Table 6:** **Effect of** *** Cinnamomum tamala *** **oil on glycosylated hemoglobin (HbA1c), hepatic glycogen and insulin**

**Treatment**	**Dose**	**HbA1c (% of Hb)**	**Hepatic glycogen (mg/g wt of tissue)**	**Insulin (micro U/ml)**
Normal	--	6 ± 1.4	75 ± 6.7	15 ± 2.2
Diabetic Control	--	10.8 ± 2.3^a^	28 ± 4.6^a^	7.8 ± 1.2^a^
CTO	100 mg/kg	9.0 ± 3.2	46 ± 2.3*	9.8 ± 2.2
CTO	200 mg/kg	7.4 ± 1.6**	62 ± 4.6**	12 ± 2.1*
Cinnamaldehyde	20 mg/kg	7.0 ± 0.6**	66 ± 2.3**	13 ± 1.6*
Glibenclamide	0.6 mg/kg	6.8 ± 0.9**	64 ± 3.4**	12.5 ± 1.8*

Values are presented as mean ± S.E.M; n = 6 in each group. One way ANOVA followed by Dunnett’s test ^a^p < 0.01 vs. normal; *p < 0.05; **p < 0.01 vs. diabetic control; *CTO*: *Cinnamomum tamala* oil.

### Effect of CTO on glycosylated hemoglobin (HbA1c)

The effect of CTO and cinnamaldehyde on HbA1c in diabetic rats is shown in the Table 6. The level of glycosylated hemoglobin significantly increased (p < 0.01) in diabetic rats as compared to normal control group. The diabetic rats when treated with CTO and cinnamaldehyde for 28 days showed a significant (p < 0.01) decreased level of glycosylated Hb as compared to untreated diabetic group. The fall in glycosylated hemoglobin level was found to be dose dependent.

### Effect of CTO on hepatic glycogen

The hepatic glycogen content in diabetic rats decreased sharply as compared to control animal (Table 6). After chronic administration of CTO and cinnamaldehyde to diabetic rats, a significant increased (p < 0.01) liver glycogen content as compared to diabetic control group was observed.

### Effect of CTO on lipid profile

Table 7 shows the level of lipids in normal and tested animals. There was a significant decrease in the level of HDL-cholesterol and a significant increase in the levels of total cholesterol and triglycerides in diabetic rats when compared to normal rats. The administration of CTO and the cinnamaldehyde reverse the level of lipids significantly (p < 0.05 and p < 0.01).

**Table 7:** **Effect of** *** Cinnamomum tamala *** **oil on Lipid profile**

**Treatment**	**Dose**	**Cholesterol (mg/dl)**	**Triglyceride (mg/dl)**	**HDL (mg/dl)**
Normal	--	84 ± 1.4	15 ± 2.6	60 ± 2.1
Diabetic Control	--	222 ± 2.3^a^	40 ± 3.2^a^	36.4 ± 1.2^a^
CTO	100 mg/kg	160 ± 3.2**	28 ± 1.8**	45 ± 3.1
CTO	200 mg/kg	100 ± 1.6**	20 ± 2.3**	52 ± 2.4*
Cinnamaldehyde	20 mg/kg	110 ± 0.6**	18.2 ± 1.7**	55 ± 1.3**
Glibenclamide	0.6 mg/kg	130 ± 0.9**	14.8 ± 1.3**	51.2 ± 1.6*

Values are presented as mean ± S.E.M; n = 6 in each group. One way ANOVA followed by Dunnett’s test ^a^ p < 0.01 vs. normal; *p < 0.05; **p < 0.01 vs. diabetic control; *CTO*: *Cinnamomum tamala* oil.

### Effect of CTO on *in vitro* antioxidant parameters

#### DPPH radical-scavenging assay

It was found that the radical- scavenging activity of CTO increased with increasing concentrations. IC_50_ for DPPH radical-scavenging activity of oil was 250 ± 1.2 μg/ml. The IC_50_ values for cinnamaldehyde, BHT and BHA were found 120 ± 0.8, 19 ± 0.1 and 18.3 ± 0.1 μg/ml respectively (Table 8).

**Table 8:** **Effect of** *** Cinnamomum tamala *** **oil and positive controls on** *** in vitro *** **Assays (DPPH, Superoxide and metal chelating Assay)**

	**IC**_**50**_**(microgram/ml)**		
**Sample**	**DPPH**	**Hydrogen peroxide (H**_**2**_**O**_**2**_**)**	**Metal chelating activity**
CTO	250 ± 1.2	180 ± 1.4	350 ± 1.3
Cinnamaldehyde	120 ± 0.8	140 ± 1.1	240 ± 0.9
BHT	19 ± 0.1	40 ± 0.2	--
BHA	18.3 ± 0.1	54 ± 0.2	--
EDTA	--	--	220 ± 0.2

*BHA*: Butylated hydroxy aniline; *BHT*: Butylated hydroxy toluene; *EDTA*: Ethylene diammine tetra acetic acid; *CTO*: *Cinnamomum tamala* oil; --: Not tested.

### H_2_O_2_ radical scavenging assay

The CTO was capable of scavenging hydrogen peroxide in a concentration- dependent manner. IC_50_ for H_2_O_2_ scavenging activity was 180 ± 1.4 μg/ml. The IC_50_ values for cinnamaldehyde, BHT and BHA were 140 ± 1.1, 40 ± 0.2 and 54.0 ± 0.2 μg/ml respectively (Table 8).

### Metal chelating ability

Tested oil and cinnamaldehyde exhibited good Fe2+ chelating ability with IC_50_ value of 350 ± 1.3 and 240 ± 0.9 μg/ml. EDTA showed very strong activity (IC50 = 220 ± 0.2 μg/ml (Table 8).

### Effect of CTO on *in vivo* antioxidant parameters

The data depicted in Table 9 indicates the effect of oil on malondialdehyde and reduced glutathione level. MDA level was found to be significantly higher in diabetic rats compared to normal rats. The oil at dose 200 mg/kg body weight p.o significantly reduced the level of MDA in diabetic rats. GSH level was found to be significantly lowered in diabetic rats as compared to normal rats. The chronic administration of CTO at 200 mg/kg body weight significantly increased the level of glutathione in diabetic rats.

**Table 9:** **Effect of** *** Cinnamomum tamala *** **oil on MDA and GSH**

**Treatment**	**Dose**	**MDA (nmol/dl)**	**GSH ( μmol GSH/g )**
Normal	--	2.7 ± 0.2	40.2 ± 2.8
Diabetic Control	--	5.2 ± 0.4 ^a^	14 ± 1.15 ^a^
CTO	100 mg/kg	4.0 ± 0.5	20 ± 2.3
CTO	200 mg/kg	3.2 ± 0.2**	32 ± 4.6**
Cinnamaldehyde	20 mg/kg	3.0 ± 0.1**	34 ± 1.1**
Glibenclamide	0.6 mg/kg	2.8 ± 0.1**	36 ± 2.8**

Values are presented as mean ± S.E.M; n = 6 in each group. One way ANOVA followed by Dunnett’s test ^a^p < 0.01 vs. normal; **p < 0.01 vs. diabetic control; CTO: *Cinnamomum tamala* oil.

## Discussion

The aim of the study was to evaluate the antidiabetic, antihyperlipidemic and antioxidant potential of the CTO and its active constituent cinnamaldehyde in STZ induced diabetes in rats. Diabetes mellitus causes a disturbance in the uptake of glucose as well as glucose metabolism. A dose of STZ as low as 50 mg/kg produces an incomplete destruction of pancreatic beta cells even though the rats become permanently diabetic [28]. After treatment with a low dose of STZ many beta cells survive and regeneration is also possible [29]. Hyperglycemia generates abnormally high levels of free radicals by autoxidation of glucose and protein glycation, and oxidative stress has been reported to be a positive factor of cardiovascular complications in STZ-induced diabetes mellitus [30]. Hyperglycemia is associated with the generation of reactive oxygen species (ROS) causing oxidative damage particularly to heart, kidney, eyes, nerves, liver, small and large vessels and gastrointestinal system [31]. The increased levels of plasma glucose in diabetic rats were lowered by CTO and cinnamaldehyde administration. The antihyperglycemic action of cinnamaldehyde results from the potentiation of insulin from existing beta cells of the islets of Langerhans [32].

To study the effect of CTO a preliminary investigation was carried out using acute antihyperglycemic model which revealed the significant reduction in glucose level. Therefore further chronic antihyperglycemic model for a period of 28 days to study the effect on various other parameters viz. insulin level, liver glycogen content, glycosylated hemoglobin, total plasma cholesterol, triglyceride and antioxidant parameters were estimated for all treated groups and compared against diabetic control group. The plasma glucose lowering activity was compared with glibenclamide, a standard hypoglycemic drug. Glibenclamide has been used for many years to treat diabetes, to stimulate insulin secretion from pancreatic beta cells [33]. From the results of the present study, it appears that still insulin producing cells are functioning and the stimulation of insulin release could be responsible for most of the metabolic effects. It may be suggested that the mechanism of action of CTO is similar to glibenclamide. The glucose lowering activity of CTO may be related to both pancreatic (enhancement of insulin secretion) and extra pancreatic (peripheral utilization of glucose) mechanism. The hyperglycemic activity was almost similar to cinnamaldehyde thereby the major constituent responsible for this activity of CTO may be cinnamaldehyde.

An increase in the level of glycosylated hemoglobin (HbA1c) in the diabetic control group of rats is due to the presence of large amount of blood glucose which reacts with hemoglobin to form glycosylated hemoglobin [34]. Oxidative stress increases due to the activation of transcription factors, advanced glycated end products (AGEs), and protein kinase C. If diabetes is persistent for long time, the glycosylated hemoglobin is found to increase [35]. The level of HbA_1_C was decreased after the administration of CTO and its main constituent cinnamaldehyde as compared to diabetic control group. The effect of cinnamon in clinical study is also reported in which the mean HbA1c was significantly decreased (P < 0.005) in the cinnamon group (8.22% to 7.86%) compared with placebo group (8.55% to 8.68%). Thus the Cinnamon supplementation could be considered as an additional dietary supplement option to regulate blood glucose level along with conventional medications to treat type 2 diabetes mellitus [36]. In diabetes mellitus, the loss of body weight is caused by increase in muscle wasting and catabolism of fat and proteins [37]. Due to insulin deficiency protein content is decreased in muscular tissue by proteolysis [38]. A decrease in body weight was registered in case of diabetic control group rats while in tested groups the weight loss was reversed. Fatty acid mobilisation from adipose tissue is sensitive to insulin. Insulin’s most potent action is the suppression of adipose tissue lipolysis [39]. A rise in plasma insulin concentration of only 5 IU/ml inhibits lipolysis by 50%, whereas a reduction in basal insulin levels result in a marked acceleration of lipolysis [40]. We demonstrated that CTO increased plasma insulin concentrations in diabetic rats. Insulin levels higher than those of the control group may result in inhibition of lipolysis and decreased plasma triglyceride and cholesterol levels. Some studies suggest that the antihyperglycemic action of traditional antidiabetic plant extracts may be due in part to decreased glucose absorption in vivo [41]. This mechanistic explanation may also apply to the actions of CTO in lowering the triglyceride and cholesterol level.

The conversion of glucose to glycogen in the liver cells is dependent on the extracellular glucose concentration and on the availability of insulin which stimulates glycogen synthesis over a wide range of glucose concentration [35]. Diabetes reduces activity of glycogen synthase thereby affecting the glycogen storage and synthesis in rat liver and skeletal muscle [27]. Oral administration of CTO 200 mg/kg body weight significantly increased hepatic glycogen levels in diabetic rats possibly because of the reactivation of the glycogen synthase system as a result of increased insulin secretion. In the clinical study the use of species of cinnamon (*Cinnamomum zeylanicum*) showed a beneficial effect on glycemic control (both HbA1c and Fating plasma glucose) and the short term (<4 months) effects of the use of cinnamon on glycaemic control looks promising [42]. The effect of *Cinnamomum zeylanicum* is also reported on gastric emptying, arterial stiffness, postprandial lipemia, glycemia, and appetite responses to high-fat breakfast [43]. Further the work can be explored for mechanism of action.

## Conclusion

In conclusion, the present study showed that oral administration of *Cinnamomum tamala* oil and its main constituent has potential antidiabetic, antihyperlipidemic and antioxidant effect in STZ induced diabetes in rats in our model systems. The potent antioxidant activity may be responsible for the antihyperglycemic and antihyperlipidemic effects. This investigation reveals the potential of CTO for use as a natural oral agent with antidiabetic, antihyperlipidemic and antioxidant effects.

## Competing interests

The authors declare that they have no competing interests.

## Authors’ contributions

SK designed and planned the study; carried out experimental work, biochemical analysis, statistical analysis, interpretation and discussion of results related to their part of the work. SS and NV designed and planned the study; drafted and revised the manuscript. NV checked and corrected the English language. All authors read and approved the final manuscript.
